# Supplementary material for: Diagnostic accuracy of the ESAT6-CFP10 skin test for latent tuberculosis infection among jail detainees
Source: Microbiol Spectr. 2025 Sep 17;13(10):e01500-25. doi: 10.1128/spectrum.01500-25 (PMC12688083; doi:10.1128/spectrum.01500-25)
Supplement: Table S1 — Diagnostic performance of the EC skin test and TST. [file spectrum.01500-25-s0001.docx]

| Appendix Table 1. Diagnostic performance of EC skin test and TST . | | | | | | | | | |  |
| --- | --- | --- | --- | --- | --- | --- | --- | --- | --- | --- |
| Parameter | TST | | AUC  (95%CI) | Sensitivity  (%, 95%CI) | Specificity  (%, 95%CI) | PPV  (%, 95%CI) | NPV  (%, 95%CI) | Kappa  (95%CI) | *P* | |
|  | Positive | Negative |  |  |  |  |  |  |  |  |
| EC |  |  |  |  |  |  |  |  |  | |
| Positive | 73 | 37 | 0.672  (0.637-0.708) | 38.8  (31.9-45.8) | 95.6  (94.3-97.0) | 66.4  (57.5-75.2) | 87.6  (85.5-89.7) | 0.411  (0.336-0.487) | 0.000 | |
| Negative | 115 | 813 |  |  |  |  |  |  |  |  |
| * NA= Not Available; PPV: Positive predictive value; NPV: Negative predictive value; Kappa coefficients were categorized as poor (κ≤ 0.20 ) , fair (0.20＜κ≤ 0.40 ) , moderate (0.40＜κ≤ 0.60 ) , good (0.60＜κ≤ 0.80 ) , and very good (0.80＜κ≤ 1.00 ) . | | | | | | | | | | |
